# Shi-Zhen-An-Shen Decoction, a Herbal Medicine That Reverses Cuprizone-Induced Demyelination and Behavioral Deficits in Mice Independent of the Neuregulin-1 Pathway

**DOI:** 10.1155/2021/8812362

**Published:** 2021-02-25

**Authors:** Chao Ma, Yan Wu, Xinyao Liu, Yi He, Yuan Jia, Pei Chen, Dongqing Yin, Yanzhe Ning, Guoqiang Xing, Zuoli Sun, Hongxiao Jia

**Affiliations:** ^1^The National Clinical Research Center for Mental Disorders & Beijing Key Laboratory of Mental Disorders, Beijing Anding Hospital, Capital Medical University, Beijing, China; ^2^Advanced Innovation Center for Human Brain Protection, Capital Medical University, Beijing, China; ^3^The Affiliated Hospital and the 2nd Clinical Medical College of North Sichuan Medical University, Nanchong Central Hospital, Nanchong 637000, China

## Abstract

Shi-Zhen-An-Shen decoction (SZASD), a Chinese herbal medicine that is a liquor extracted from plants by boiling, has been reported to be effective in treating schizophrenia. However, the mechanism is unclear. Abnormal demyelination has been implicated in schizophrenia. The aim of this study was to investigate the effect of SZASD on myelin in demyelinated mice exhibiting schizophrenia-like behaviors. Sixty male C57BL/6 mice were randomly divided into six groups (*n* = 10 per group): (1) control group, (2) cuprizone (CPZ, a copper chelator that induced demyelination, 0.2% *w*/*w*)+saline, (3) CPZ+low-dose SZASD (8.65 g·kg^−1^·d^−1^), (4) CPZ+medium-dose SZASD (17.29 g·kg^−1^·d^−1^), (5) CPZ+high-dose SZASD (25.94 g·kg^−1^·d^−1^), and (6) CPZ+quetiapine (QTP, an atypical antipsychotic that served as a positive treatment control, 10 mg·kg^−1^·d^−1^). Mice in groups 2-6 were treated with CPZ added to rodent chow for six weeks to induce demyelination. During the last two weeks, these mice were given an oral gavage of sterile saline, SZASD, or quetiapine. Behavioral tests and brain analyses were conducted after the last treatment. The brain expression of myelin basic protein (MBP) and neuregulin-1 (NRG-1) was assessed using immunohistochemistry and Western blots. CPZ induced significant schizophrenia-like behaviors in the mice, including reduced nest-building activity and sensory gating deficits. Hyperlocomotor activity was accompanied by significant reductions in MBP expression in the corpus callosum, hippocampus, and cerebral cortex. However, both QTP and SZASD significantly reversed the schizophrenia-like behaviors and demyelination in CPZ-fed mice. The QTP and medium-dose SZASD resulted in better therapeutic effects compared to the low and high SZASD doses. Reduced NRG-1 expression was observed in CPZ-fed mice compared with controls, but neither QTP nor SZASD showed significant influence on NRG-1 expression in the hippocampus. Together, SZASD showed a therapeutic effect on demyelinated mice, and the improvement of demyelination might not be through the NRG-1 pathway.

## 1. Introduction

Schizophrenia is a severe, debilitating neuropsychiatric disorder affecting about 1% of the population worldwide [1]. The lifetime prevalence of schizophrenia is approximately 0.6% in China [2]. Schizophrenia is associated with long-term disabilities, considerable economic burden, and challenging social responsibility. Clinical symptoms of schizophrenia are classified as positive (e.g., hallucinations and delusions), negative (e.g., emotional blunting and social withdrawal), and cognitive deficits (problems with attention, processing speed, and working memory) [3].

Antipsychotic therapy is the primary clinical treatment for schizophrenia. Although classical and atypical antipsychotics can be able to reduce delusions and hallucinations, they have little effect on negative symptoms and cognitive impairment exhibited by schizophrenia patients [4]. Therefore, alternative treatments for schizophrenia are needed. We have prescribed Shi-Zhen-An-Shen decoction (SZASD), an empirical Chinese herb prescription for individuals at extreme risk for psychosis at Beijing Anding Hospital, and reported significantly relieved psychiatric symptoms and improved cognitive function in schizophrenia patients [5]. Also, numerous reports have described divergent therapeutic effects for the active components of SZASD, including cornel iridoid glycoside(CIG) [6] and tetrahydroxystilbene glucoside(TSG) [7], on neurological defects and cognitive impairment. Although increasing evidence supports the neuroprotective effects of SZASD on neurological disorders, its mechanisms of action remain unclear.

Although the interaction of genetics and environmental factors is thought to be involved in the development of schizophrenia, the specific pathophysiological processes involved in the disease development and progression are largely unknown. Many studies have focused on the role of changes in gray matter in the pathogenesis of schizophrenia. Other studies, including magnetic resonance imaging (MRI) [8] and genetic analysis [9], suggest that abnormalities in white matter (WM) and myelin sheaths are involved in the etiopathology of schizophrenia. The myelin sheaths in the central nervous system (CNS) are primarily composed of oligodendrocytes and function to preserve axonal integrity for rapid and efficient conduction of the electrical impulses along axons [10]. The loss of myelin may result in axonal degeneration and neuronal dysfunction, which leads to cognitive deficits [11].

Cuprizone (CPZ) is a neurotoxic agent that acts as a copper chelator [12], causing damage to the oligodendrocytes [13]. Studies that fed mice with 0.2% (*w*/*w*) CPZ for several weeks induced chronic demyelination. Recent imaging studies showed that there was significant demyelination in the corpus callosum of mice fed with 0.2% CPZ for six weeks [14]. Mice also exhibited abnormal behaviors, including impaired sensory gating [15] and impaired memory [16] when exposed to CPZ for three or four weeks. Quetiapine (QTP) (an atypical antipsychotic) significantly improved the schizophrenia-like behaviors and reduced myelin loss in CPZ-fed mice [17, 18]. Previous studies revealed that QTP at the dose of 10 mg/kg could attenuate some of the changes observed in CPZ-fed rats [19, 20].

The mechanisms of myelin development, degeneration, and regeneration are complex. Considerable evidence shows that neuregulin- (NRG-) 1 plays an important role in regulating the myelination process [21]. Furthermore, clinical [22] and animal studies [23] indicate a critical role of NRG-1 in the development of schizophrenia. Thus, we speculated that NRG-1 was a potential target for the therapeutic effect of SZASD on schizophrenia. This study examined the effects of SZASD on schizophrenia-like behaviors in mice in which demyelination was induced by exposure to CPZ. We hypothesized that SZASD would exert its antipsychotic effects by protecting the myelin sheath through the NRG-1 signaling pathway.

## 2. Materials and Methods

### 2.1. Animals

Sixty six-week-old male C57BL/6 mice weighing 20 ± 2 g were used. Mice were obtained from the laboratory animal center at Capital Medical University. They were housed in SPF environment with a 12 h light/dark cycle and a temperature- and humidity-controlled facility (22 ± 1°C and relative humidity of 55% to 60%), with free access to food and water. All animal experiments were approved by the Institutional Animal Care and Use Committee of Capital Medical University (AEEI-2018-047).

### 2.2. Drugs

CPZ (Sigma-Aldrich, St. Louis, MO, USA) was added to the rodent chow with a final concentration of 0.2% (*w*/*w*). As a positive control, additional mice were treated with quetiapine (AstraZeneca, Wilmington, DE, USA, 10 mg·kg^−1^·d^−1^, QTP), a widely used antipsychotic [18]. SZASD is a traditional Chinese herb formula, which is composed of *Chrysanthemum* (菊花), *Rehmannia glutinosa* (干地黄), and *Polygoni Multiflori Radix* (何首乌) and other components [5]. All herbs were purchased from Beijing Tong Ren Tang (Group, Co., Ltd., Beijing, China). The mixed herbs were boiled at 100°C in an appropriate volume for 1 h, and the extraction procedure was repeated twice. The aqueous extracts were filtered, combined, and further concentrated using rotary evaporation under vacuum in a 60°C water bath. The concentrated extract was lyophilized to create a SZASD powder (yield: 32.0%) and stored under desiccation at room temperature.

### 2.3. Drug Preparation and Administration

The dose of SZASD administered to the mice was increased 9.1-fold compared to the typical human dose, based on the formula for the body surface area. For an average adult human weighing 70 kg, the typical dose is 1.9 g herbs·kg^−1^·d^−1^. The low (L), medium (M), and high (H) doses for mice were calculated as 8.65, 17.29, and 25.94 g ·kg^−1^·d^−1^, respectively. The powder was dissolved in sterile saline (NS) before administration via gavage. The final volume used for gavage feeding was 0.06 mL/10 g. The control and QTP-treated groups were gavaged daily with an equal volume of sterile saline.

### 2.4. Experimental Design

**Figure 1:**
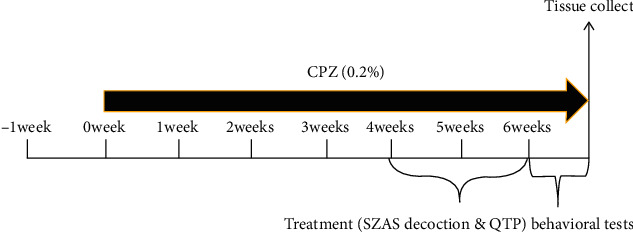
Study design. Mice in the control group were fed with regular rodent chow, while the cuprizone exposure groups were fed a diet containing 0.2% cuprizone. Behavioral assessments were conducted by one or two trained observers on the indicated day throughout the entire study. All mice were euthanized when behavioral tests were completed.

Sixty mice were randomly divided into six groups (*n* = 10 per group). Mice in group 1, the control group (control), received regular rodent chow and tap water. Group 2 mice (CPZ+NS) were fed a rodent chow containing CPZ from weeks zero to six (10 mg·kg^−^·d^−1^) to induce chronic demyelination. The CPZ was administrated with NS intragastrically via gavage during the last two weeks of treatment. Group 3 mice (CPZ+L(SZASD)) that received a low dose of SZASD (8.65 g·kg^−1^·d^−1^), group 4 mice (CPZ+M(SZASD)) that received a medium dose of SZASD (17.29 g·kg^−1^·d^−1^), group 5 mice (CPZ+H(SZASD)) that received a high dose of SZASD (25.94 g·kg^−1^·d^−1^), and group 6 mice (CPZ+QTP) that received QTP (10 mg·kg^−1^·d^−1^) were treated with rodent chow containing 0.2% CPZ (*w*/*w*) during weeks zero through six [24]. SZASD and QTP were administered using oral gavage once daily for two weeks (Figure 1). The mice were weighed weekly.

### 2.5. Behavioral Tests

The behavioral tests were conducted 24 hours after the completion of the two-week drug exposure.

#### 2.5.1. Nest-Building Activity

As previously described, each mouse was individually housed in a new cage overnight with access to food and water ad libitum. One unit of pressed cotton batting (5 cm × 5 cm) was placed in a corner of the cage [25]. Normal mice perform typical nest-building activities in the “home” cage. A five-point criteria system was used to evaluate the nest-building activity of mice the following morning [26]. The criteria included the following: 1 = pressed cotton batting was scattered throughout the cage, not bitten; 2 = pieces of pressed cotton batting were applied to a side of the cage, but relatively loose and not forming a nest, and with no obvious tearing or folding; 3 = pieces of pressed cotton batting were folded together to form a nest, but the nest was relatively flat and did not exhibit any visible tearing; 4 = pieces of the pressed cotton batting were folded together to form a ball-shaped nest that covered the mouse, and the batting was bitten into small pieces; and 5 = pieces of the pressed cotton batting were folded together to form a ball-shaped nest that covered the mouse, and the nest walls were higher than the height of the mouse's body. The nest-building scores were obtained blindly by the same observer [27].

#### 2.5.2. Open-Field Test

The open-field test was used to measure the locomotor activity, exploratory behavior, and anxiety-related behavior of the mice. Tests were performed in a quiet house with low light levels. The day before the experiment, the mice were moved to this room to allow them to adapt to the environment. The open-field test was performed in a square box (40 × 40 × 25 cm) painted gray. A video camera was placed 1.5 meters above the box. The camera was connected to a computer equipped with the Supermaze System (Shanghai Xinruan Information Technology, Co. Ltd., Shanghai, China) to evaluate locomotor activity. Each mouse was placed in the center of the box and tested individually in the open field for 30 min, and the behaviors were recorded. After each test, the equipment was cleaned with 70% alcohol. The total distance traveled and time spent at the perimeter were analyzed.

#### 2.5.3. Prepulse Inhibition (PPI)

PPI was performed according to published methods with slight modifications [28]. The mice were exposed to a series of startle pulses with or without a short acoustic prepulse. Auditory startle reflex and sensory gating were evaluated using commercial startle chambers (MED-ASR-PRO1, MED Associates Inc., USA). White noise (60 dB) was provided as the background noise, and the experiment was divided into three stages, including an adjustment period, block I, and block II. After an adjustment period of five minutes, block I was set to 20 trials at 20 s intervals. Each trial included a single 20 ms 110 dB startle pulse stimulus with no acoustic prepulse. Block II included 60 trials at 20 s intervals with an acoustic prepulse and co5vered six types of stimuli, including a 2 ms duration prepulse at 75 dB or 85 dB, followed by no-startle stimulus or a 20 ms 110 dB startle stimulus with a 30 ms or 100 ms delay. Each trial type was randomly repeated ten times. The PPI score was calculated using the block II data and the following formula: PPI = (1 − prepulse plus startle amplitude/startle only amplitude) × 100. PPI sessions were performed between 08:00 and 12:00 a.m.

### 2.6. Biochemical Analyses

#### 2.6.1. Tissue Preparation

After the behavioral tests were completed, five mice from each group were used for immunohistochemical staining, and the other five mice were used for Western blot analysis. The mice used for immunohistochemical staining were deeply anesthetized using pentobarbital sodium (250 mg/kg, intraperitoneal injection (i.p.)) and then transcardially perfused with saline, followed by 4% paraformaldehyde in PBS (0.1 M, pH =7.4) [29]. The brains were removed and immersed in the same fixative overnight at 4°C, followed by cryoprotection in 20% sucrose for 24 h and 30% sucrose for 48 h, both at 4°C. The brains were cut into serial coronal sections (30 *μ*m) using a sliding microtome, and the sections were collected into 16-well plates for cryopreservation. The mice used for Western blots were euthanized using cervical dislocation. The hippocampus and cerebral cortex were removed from the brains and quickly stored at -80°C for Western blot analysis.

#### 2.6.2. Immunohistochemical Staining

Free-floating sections were placed in PBS and washed three times at room temperature (RT) and then permeabilized with 0.3% Triton for 30 min at RT. Then, the sections were washed three times in PBS and incubated in PBS with 10% hydrogen peroxide for 30 min at RT. After washing three times with PBS, the sections were incubated with goat serum for 30 min at RT to block nonspecific antigens. Subsequently, the sections were incubated in a solution containing an MBP (myelin basic protein) rabbit polyclonal primary antibody (1 : 200 dilution, Sigma-Aldrich, St. Louis, MO, USA) overnight at 4°C. After rinsing in PBS, the sections were incubated with a secondary antibody conjugated with HRP (Zhongshan Golden Bridge Biology Company, Beijing, China) for 20 min at RT. The antigen-antibody complexes were visualized using 0.025% diaminobenzidine (DAB; Zhongshan Golden Bridge Biology Company, Beijing, China) as the chromogen. All sections were washed in PBS and mounted on amino propyltriethoxysilane-coated slides. The mounted sections were dehydrated using a graded series of ethanol, cleared in xylene, and coverslipped with immunohistochemical sealed with water-soluble tablets. Images were obtained using a Nikon BX-51 light microscope and analyzed using Image-Pro Plus 6 (Media Cybernetics, Inc., Bethesda, MD, USA).

#### 2.6.3. Western Blot Analysis

The proteins were extracted with Tris-EDTA lysis buffer (1 mM EDTA, 20 mM Tris, pH 7.5, 1% Triton X-100, and 10% glycerol) containing a protease inhibitor cocktail (Sigma-Aldrich, St. Louis, MO, USA). After the content was determined, the proteins were separated by sodium dodecyl sulfate-polyacrylamide gel electrophoresis and transferred onto Immobilon-FL NC membranes (Millipore UK Ltd., Watford, UK). Membranes were blocked with Tris-buffered saline containing 0.1% Tween-20 and 5% nonfat milk (PBST/5% milk) and then probed with specific antibodies at 4°C overnight. Membranes were then washed with PBST and incubated for 1 or 2 h in PBST/5% milk containing horseradish peroxidase- (HRP-) conjugated secondary antibodies (1 : 2000, Zhongshan Golden Bridge Biology Company, Beijing, China); then, the target band was focused through enhanced chemiluminescence (ECL System, Bio-Rad, Hercules, CA, USA). Images were captured by the V3 Workflow (Bio-Rad, Hercules, CA, USA), and the bands visualized in the gels were analyzed using the Image Lab Software (Media Cybernetics, Inc., Bethesda, MD, USA). The following primary antibodies were used in this study: rabbit monoclonal anti-MBP (1 : 1000, Abcam, Cambridge, UK), rabbit polyclonal anti-NRG1 (1 : 1000, Abcam, Cambridge, UK), and mouse monoclonal anti-*β*-actin antibody (1 : 1000; Santa Cruz, Dallas, Texas, USA).

### 2.7. Statistical Analysis

SPSS software version 20 was used for data analysis, and GraphPad software (Prism version 6) was used for charting. The data in the statistical chart were expressed as the means ± SEM (Standard Error of Mean). The difference of PPI and nest-building ability among groups was analyzed by the Wilcoxon rank-sum test. Repeated measures ANOVA with Bonferroni adjustment was used to compare the weight of mice among groups. The other data were analyzed using one-way analysis of variance (ANOVA), and Dunnett's *post hoc* test was used for multiple comparisons. The *p* value of less than 0.05 was considered significant.

## 3. Results

### 3.1. Body Weight Changes

All mice exhibited shiny, smooth fur, as well as normal activity levels and feeding behavior. No mice died during the experiment. Repeated measures ANOVA for body weight showed that there was a significant main effect for time (*F*_time_ = 675.87, *p*_time_ < 0.001), group assignment (*F*_group_ = 31.19, *p*_group_ < 0.001), and interaction (*F*_group_ = 28.43, *p*_interaction_ < 0.001), meaning a significant difference in body weight from baseline to the end of 6 weeks among groups (Table 1). The control group showed sustained body weight gain during the experimental period. The CPZ-treated mice exhibited lower body weight gain than the control mice, starting in the second week of the experiment. As shown in Table 1, both medium-dose SZASD and QTP remarkably increased the body weight of CPZ-treated mice from the 4^th^ week to the 6^th^ week.

### 3.2. Schizophrenia-Like Behaviors

The CPZ-treated mice presented schizophrenia-like behaviors, including abnormal nesting activity, locomotor activity, and PPI.

**Figure 2:**
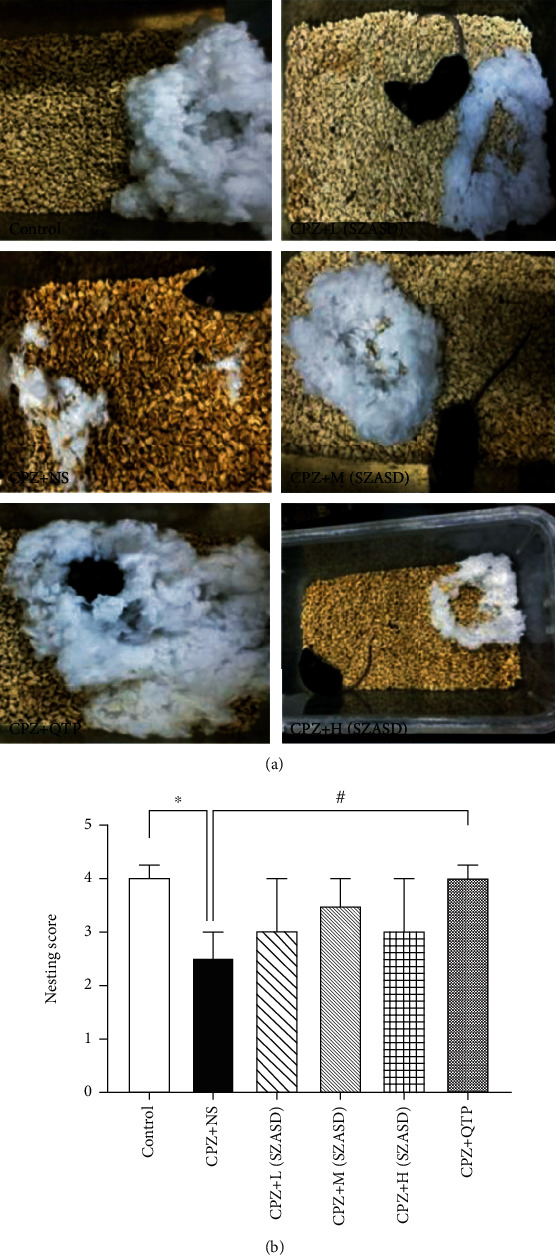
Nesting activity was altered by exposure to cuprizone and partially rescued by quetiapine and SZASD. (a) Representative images of nesting activity of mice in the control, CPZ, CPZ+L(SZASD), CPZ+M(SZASD), CPZ+H(SZASD), and CPZ+QTP groups. (b) Histogram of nesting scores in mice among the six different groups. Values are expressed as median (interquartile range) (*n* = 10, ^∗∗^*p* < 0.01, model group vs. control group; ^#^*p* < 0.01, ^##^*p* < 0.01, drug groups vs. model group). Legend: CPZ+L(SZASD): 8.65 g·kg^−1^·d^−1^; CPZ+M(SZASD): 17.29 g·kg^−1^·d^−1^; CPZ+H(SZASD): 25.94 g·kg^−1^·d^−1^; CPZ+QTP: 10 mg·kg^−1^·d^−1^.

A significant decrease in nest-building scores was observed in the CPZ+NS group compared to the naive controls (*p* < 0.01, Figure 2). CPZ-fed mice treated with three different doses of SZASD did not show significant changes in nest-building scores compared with the CPZ+NS group (all *p* > 0.05), although QTP significantly increased the nest-building ability in CPZ-fed mice (*p* < 0.05).

**Figure 3:**
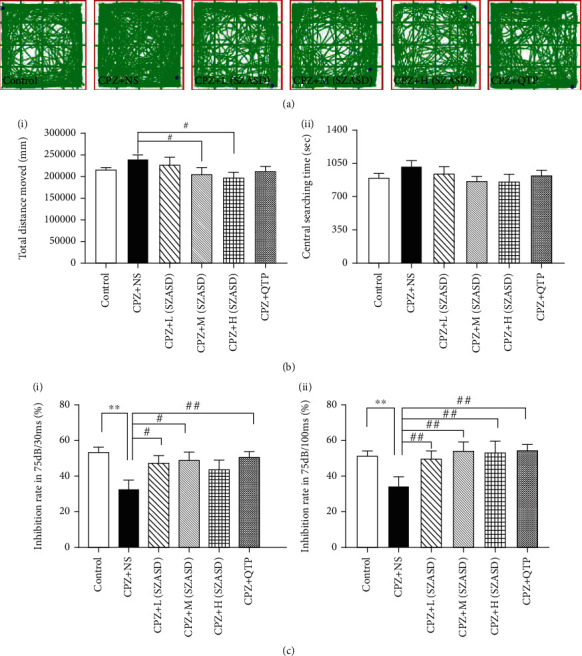
Locomotor activity and PPI deficits due to exposure to cuprizone rescued by quetiapine and SZASD. (a) Representative movement traces of mice in the control, CPZ+NS, CPZ+L(SZASD), CPZ+M(SZASD), CPZ+H(SZASD), and CPZ+QTP groups. (b) (i) Comparison of total locomotor activity (total movement distance in 30 min) of mice among groups. (ii) Comparison of center zone movement distance in 30 min of mice among groups. (c) Comparison of (i) PPI at 75 dB with a 30 ms interval and (ii) PPI at 75 dB with a 100 ms interval among groups. Values are expressed as mean ± SEM (Standard Error of Mean) (*n* = 10-14, ^∗∗^*p* < 0.01, model group vs. control group; ^#^*p* < 0.01, ^##^*p* < 0.01, drug groups vs. model group). Legend: CPZ+L(SZASD): 8.65 g·kg^−1^·d^−1^; CPZ+M(SZASD): 17.29 g·kg^−1^·d^−1^; CPZ+H(SZASD): 25.94 g·kg^−1^·d^−1^; CPZ+QTP: 10 mg·kg^−1^·d^−1^.

The open-field test evaluated the locomotor activity of the mice. The CPZ+NS group traveled a relatively greater total distance than the control group (*p* > 0.05) and a significantly greater total distance than the CPZ+M-SZASD and CPZ+H-SZASD groups (*p* < 0.05, each) (Figure 3). No significant difference was observed in the time spent in the center of the open-field chamber (a marker of anxiety) among the six groups, although the CPZ+NS group showed a trend towards increased time compared to other groups.

PPI evaluates the sensory gating function, which is impaired in schizophrenia. The CPZ+NS group showed significantly lower PPIs than other groups at both the prepulse intensity of 75 dB with a 30 ms delay (*p* < 0.05, for all groups except the CPZ+H-SZASD group) and at 75 dB with a 100 ms delay (*p* < 0.01, all groups) (Figure 3(c)).

### 3.3. Myelin Sheath

**Figure 4:**
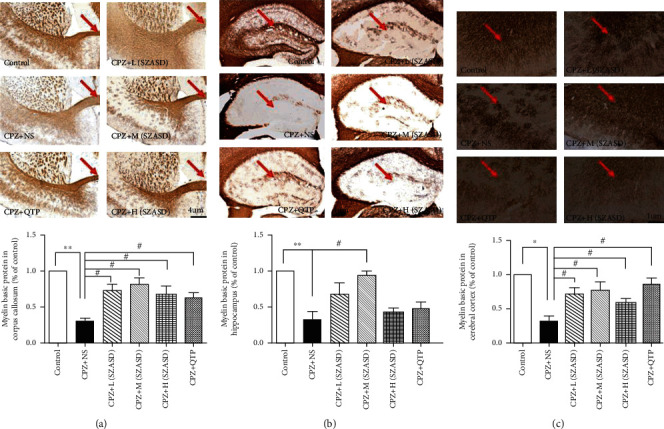
Decrease in MBP expression as visualized using immunostaining in mice induced by exposure to cuprizone and rescued by quetiapine and SZASD. (a) Corpus callosum (×40). (b) Hippocampus (×40). (c) Cerebral cortex (×100). Values are expressed as mean ± SEM (Standard Error of Mean) (*n* = 3-4; ^∗^*p* < 0.05, ^∗∗^*p* < 0.01, model group vs. control group; ^#^*p* < 0.01, ^##^*p* < 0.01, drug groups vs. model group).

**Figure 5:**
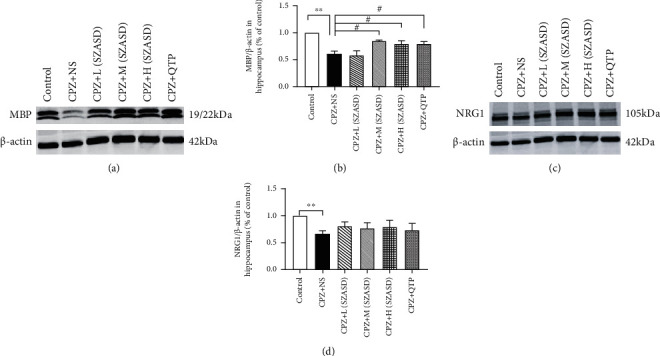
Effects of SZASD on MBP and NRG1 expression in CPZ-treated mice. (a, b) Western blot and quantitative analysis of expression of MBP protein in the hippocampus. (c, d) Western blot and quantitative analysis of expression of NRG1 protein in the hippocampus. *β*-Actin was used as an internal control. Values are expressed as mean ± SEM (Standard Error of Mean) (*n* = 4-6, ^∗∗^*p* < 0.01, model group vs. control group; ^#^*p* < 0.01, drug groups vs. model group).

MBP is the main component of myelin and is essential to maintain the structural stability and function of the myelin sheath. Immunohistochemistry analysis (Figure 4) revealed that the expression of MBP was significantly downregulated in the corpus callosum (Figure 4(a), *p* < 0.01), hippocampus (Figure 4(b), *p* < 0.01), and cerebral cortex (Figure 4(c), *p* < 0.05) after CPZ exposure, and this was reversed in mice treated with the three doses of SZASD and QTP, except for the hippocampus where the L-SZASD and H-SZASD treatments did not reverse the CPZ-induced MBP downregulation (*p* > 0.05). Western blot analysis also showed significantly decreased MBP expression in the hippocampus after CPZ exposure (*p* < 0.01) (Figures 5(a) and 5(b)) that was reversed after M-SZASD, H-SZASD, and QTP treatment, but not after L-SZASD treatment (*p* < 0.05).

### 3.4. NRG1 Expression

NRG-1 signaling is crucial for normal oligodendrocyte development and CNS myelination. Western blot analysis revealed that exposure to CPZ significantly decreased NRG-1 expression in the mouse hippocampus (Figures 5(c) and 5(d), *p* < 0.01), which was not significantly reversed after SZASD and QTP treatments, all *p* > 0.05).

## 4. Discussion

We evaluated the remyelinating properties of SZASD in CPZ-treated mice. SZASD improved schizophrenia-like behaviors in CPZ-treated mice, including sensory gating and locomotor activity. SZASD also prevented demyelination and reversed MBP loss in the cerebral cortex, corpus callosum, and hippocampus but not the loss of hippocampal NRG-1 protein of CPZ-treated mice. These results suggested that SZASD might ameliorate schizophrenia-like behavior and demyelination in CPZ-treated mice independent of the NRG-1 pathway.

Several kinds of animal models were commonly used in schizophrenia, but the pathological mechanisms are different. Common drugs used in pharmacological models are amphetamine or methamphetamine (dopamine receptor agonists), phencyclidine (PCP), and diphenhydramine maleate (MK-801), which resulted in schizophrenia-like symptoms in animals [30]. However, these animal models only showed part of the phenotypes of schizophrenia. For example, amphetamine only induces psychotic-like changes but does not mimic the negative or cognitive symptoms seen in schizophrenia [31]. MK-801 induces two main psychomotor behaviors: positive symptoms (rapid movement [32, 33]) and negative symptoms (stereotyped behavior [34]), as well as long-term impairment in associative memory and spatial working memory [35]. Studies with neuroimaging [36], gene [37, 38], and postmortem [39] showed that there was white matter damage in schizophrenia patients and animal models. Previous studies have shown that CPZ effectively induces demyelination [40] and that the demyelination process can lead to the development of schizophrenia-like behaviors [28, 41]. CPZ-fed mice exhibited loss of body weight [42], increased locomotor activity [41], impaired sensory gating [15], and loss of MBP in the brain, which were in line with our present result in this study. The hyperactivity of CPZ-fed mice in the open-field test might reflect the abnormal activity in the mesolimbic and nigrostriatal dopamine system [43]. The decreased nest-building activity in demyelinated mice might reflect self-neglect and social withdrawal [27], whereas the decline in PPI reflected impaired sensory gating, which was predominantly regulated by the cortico-striato-pallido-pontine (CSPP) system [44]. Importantly, antipsychotics such as quetiapine [18] and olanzapine [45] significantly improved the abnormal behavior and myelin sheath loss in CPZ-fed animals. Thus, exposure to CPZ offers a promising model to study the effects of WM impairment in schizophrenia.

In this study, the CPZ-fed mice exhibited significant loss of myelin in the corpus callosum, hippocampus, and cerebral cortex, which was similar to a previous study [46]. MBP is the primary component of myelin, a critical component in maintaining the integrity of the myelin sheath, and is a biomarker for axon myelination sheaths. The myelin sheath is essential for providing nutrition to the axon and maintaining the proper conduction velocity of action potentials necessary for physiological function [47]. The corpus callosum is the largest connectivity component in the brain and transfers information between the two cerebral hemispheres [48]. The corpus callosum is responsible for processing cognitive information [43]. The hippocampus is responsible for learning and memory. Imaging studies have revealed impaired WM in these brain regions of patients with schizophrenia [49]. Reduced cellular density and morphological abnormalities of oligodendrocytes have been reported in the brains of schizophrenia patients, primarily in the corpus callosum [50], anterior cingulate cortex, prefrontal cortex [51–53], and hippocampus [54]. Therefore, demyelination in the cerebral cortex, corpus callosum, and hippocampus in our study might be related to the schizophrenia-like behaviors observed in CPZ-treated mice.

To explore the antipsychotic effect of SZASD, we completed a two-arm clinical study that used the Global Assessment of Functioning Scale, Positive and Negative Syndrome Scale, and Structured Interview for Prodromal Syndromes in 54 individuals with ultrahigh risk for psychosis [5]. This study revealed that a twelve-week treatment using aripiprazole and SZASD cotherapy (vs. aripiprazole (5-10 mg/day) with a SZASD placebo) significantly improved the psychotic symptoms and cognitive deficits of the patients in their performance in the verbal learning, visual memory, continuous performance, and stroop (color/word) tests (*p* < 0.05), whereas aripiprazole monotherapy only increased the verbal learning and the stroop word test scores (*p* < 0.05). Our present study also indicated the antipsychotic effect of SZASD in CPZ-fed mice. The medium dose of SZASD significantly improved behavioral abnormalities and MBP expression, and this was similar to the effect of QTP. The low dose of SZASD only partially improved CPZ-induced neurobiological and behavioral deficits and was less effective than the medium dose of SZASD. The high dose of SZASD only moderately improved the behavioral deficits, suggesting a sedative or hypnotic effect of SZASD at the highest dose. Nevertheless, all SZASD doses used in this study appeared to be within safe and effective ranges (Supplementary material Figure 1).

Previous studies have supported the possibility of neuroprotective effects produced by the main components of SZASD in neurological diseases. For example, intragastric administration of CIG, the primary component extracted from *Rehmannia glutinosa*, ameliorated the neurological defects and cognitive impairment in rats after traumatic brain injury or fimbria-fornix transection [55]. Moreover, TSG, the main active component extracted from *Polygoni Multiflori Radix*, showed neuroprotective effects and improved learning and memory in both normal and neurotoxin-injured animals [56]. Our present results confirmed that intragastric treatment with SZASD for 14 consecutive days significantly improved schizophrenia-like behavioral deficits in CPZ-treated mice, including sensory gating and locomotor activity. Although the exact mechanism of the neuroprotective effect of SZASD is unknown, it might involve the inhibition of neuronal apoptosis and the promotion of neuroregeneration mediated by neurotrophic factors [6, 55]. Our study was the first to demonstrate the protective effect of SZASD on myelin sheath in the cerebral cortex, corpus callosum, and hippocampus and its association with improved psychological behaviors and cognitive deficits in CPZ-induced demyelinated mice.

Although NRG-1 protein has been implicated in neurodevelopment, myelination, and schizophrenia [57, 58], a definite role for NRG-1 in schizophrenia remains questionable. Both overexpression [59] and knockout NRG-1 signaling [60] have induced schizophrenia-like behaviors in animals. Furthermore, both elevated [61] and reduced [62] NRG1 levels were reported in patients with schizophrenia. In this study, the CPZ induced a significant decrease in NRG-1 protein in the hippocampus that was not reversed with SZASD (*p* > 0.05). This result suggested that NGR-1was not involved in the neuroprotective effect of SZASD-mediated remyelination and behavioral improvement in CPZ-treated mice.

Several limitations of this study should be noted. Firstly, due to the complexity of the symptoms and etiology of schizophrenia, it is difficult to replicate the full spectrum of symptoms and etiology in animal models. CPZ-fed mice only simulate symptoms related to myelin loss. Secondly, the pathophysiological process in this particular mouse model of schizophrenia is unclear and may not be specific to schizophrenia. Myelin impairment has been observed in other disorders [63], including bipolar disorder (BD), major depressive disorder (MDD), and multiple sclerosis (MS). Thirdly, the demyelination induced by CPZ may be transient. Withdrawal of CPZ can spontaneously initiate myelin repair [64]. Therefore, clinical studies and more appropriate animal models are needed to fully understand the potential mechanism of action of SZASD in schizophrenia.

## 5. Conclusions

In summary, this study demonstrated that SZASD improved schizophrenia-like behaviors and demyelination impairment in mice exposed to CPZ. However, further studies are needed to determine the biological mechanisms that underlie the therapeutic effect of SZASD.

## Figures and Tables

**Table 1:** Effects of SZASD and QTP on the body weight in CPZ-treated mice.

Group	Week 0	Week 1	Week 2	Week 3	Week 4	Week 5	Week 6
Control	22.36 ± 0.93	24.75 ± 0.62	26.00 ± 0.60	27.05 ± 0.59	27.93 ± 0.61	28.81 ± 0.76	29.61 ± 0.75
CPZ+NS	22.83 ± 0.52	24.18 ± 0.55	23.61 ± 0.59^∗∗∗^	22.83 ± 0.55^∗∗∗^	23.65 ± 0.54^∗∗∗^	24.60 ± 0.67^∗∗∗^	26.00 ± 0.74^∗∗∗^
CPZ+L(SZASD)	22.88 ± 0.67	24.51 ± 0.69	23.68 ± 0.73^∗∗∗^	23.03 ± 0.69^∗∗∗^	24.14 ± 0.76^∗∗∗^	25.48 ± 0.96^∗∗∗^	26.65 ± 1.11^∗∗∗^
CPZ+M(SZASD)	22.85 ± 0.51	24.68 ± 0.86	24.11 ± 0.80^∗∗^	23.40 ± 0.81^∗∗∗^	24.58 ± 0.71^∗∗∗^	25.96 ± 0.58^∗∗∗^^##^	27.12 ± 0.68^∗∗∗^^#^
CPZ+H(SZASD)	22.90 ± 0.69	24.53 ± 0.91	23.84 ± 0.85^∗∗∗^	23.21 ± 0.84^∗∗∗^	24.33 ± 0.81^∗∗∗^	25.31 ± 0.96^∗∗∗^	26.18 ± 0.73^∗∗∗^
CPZ+QTP	23.23 ± 0.17	25.21 ± 0.81	24.57 ± 0.90^∗∗^	24.08 ± 0.71^∗∗∗^^##^	25.05 ± 0.72^∗∗∗^^##^	26.36 ± 0.84^∗∗∗^^##^	27.70 ± 0.85^∗∗∗^^##^

Data represent mean ± SD. *n* = 12 per group. ^∗∗^*p* < 0.01, ^∗∗∗^*p* < 0.001 compared with control group; ^#^*p* < 0.05, ^##^*p* < 0.01 compared with CPZ+NS group.

## Data Availability

The original data of this study are available from the corresponding authors upon reasonable request.

## Supplementary Materials

Supplementary Materials Supplementary Figure 1: safety effects (hematoxylin and eosin (HE) staining) of SZASD with different extraction processes on different organs in female rats (180-220 g) at high doses (23.25 g·kg^−1^·d^−1^) for 26 weeks (HE, ×100). (A) Liver in the control group. (B) Liver in the high-dose group. (C) Lung in the control group. (D) Lung in the high-dose group. (E) Heart in the control group. (F) Heart in the high-dose group. (G) Kidney in the control group. (H) Kidney in the high-dose group. (I) Spleen in the control group. (J) Spleen in the high-dose group.
