# Supplementary material for: Shi-Zhen-An-Shen Decoction, a Herbal Medicine That Reverses Cuprizone-Induced Demyelination and Behavioral Deficits in Mice Independent of the Neuregulin-1 Pathway
Source: Neural Plast. 2021 Feb 25;2021:8812362. doi: 10.1155/2021/8812362 (PMC7932787; doi:10.1155/2021/8812362)
Supplement: Supplementary Materials — Supplementary Figure 1: safety effects (hematoxylin and eosin (HE) staining) of SZASD with different extraction processes on different organs in female rats (180-220 g) at high doses (23.25 g·kg−1·d−1) for 26 weeks (HE, ×100). (A) Liver in the control group. (B) Liver in the high-dose group. (C) Lung in the control group. (D) Lung in the high-dose group. (E) Heart in the control group. (F) Heart in the high-dose group. (G) Kidney in the control group. (H) Kidney in the high-dose group. (I) Spleen in the control group. (J) Spleen in the high-dose group. [file 8812362.f1.docx]

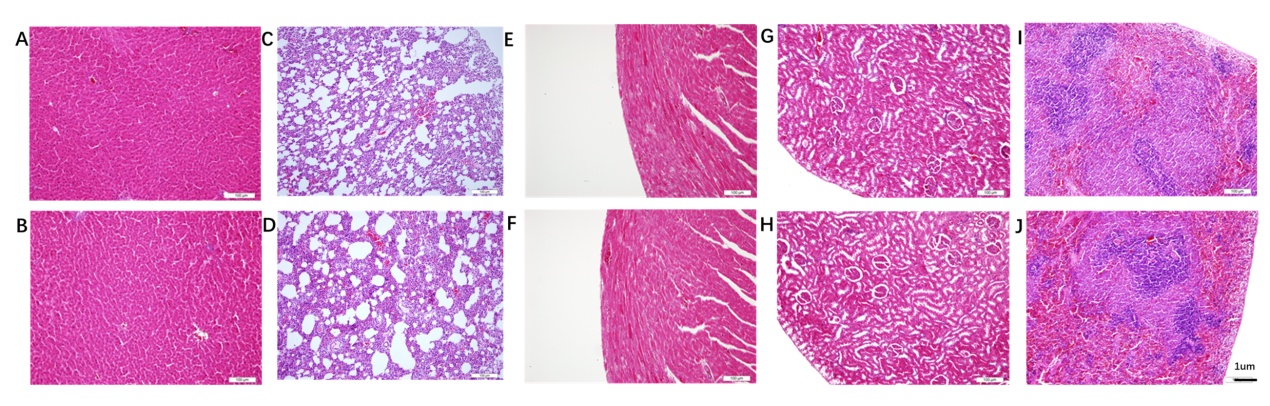


**Supplementary Figure 1.** Safety effects (hematoxylin and eosin (HE) staining) of SZASDwith different extraction processes on different organs in female rats (180-220 g) at high doses (23.25 g·kg^-1^·d^-1^) for 26 weeks. (HE, ×100). **(A)** liver in the control group. **(B)** liver in the high-dose group. **(C)** lung in the control group. **(D)** lung in the high-dose group. **(E)** heart in the control group. **(F)** heart in the high-dose group. **(G)** Kidney in the control group. **(H)** Kidney in the high-dose group. **(I)** spleen in the control group. **(J)** spleen in the high-dose group.
